# The Reversible Increase in Tight Junction Permeability Induced by Capsaicin Is Mediated via Cofilin-Actin Cytoskeletal Dynamics and Decreased Level of Occludin

**DOI:** 10.1371/journal.pone.0079954

**Published:** 2013-11-18

**Authors:** Tomoko Shiobara, Takeo Usui, Junkyu Han, Hiroko Isoda, Yoko Nagumo

**Affiliations:** Faculty of Life and Environmental Sciences, University of Tsukuba, Tsukuba, Ibaraki, Japan; University of Illinois at Chicago, United States of America

## Abstract

Previous results demonstrated that capsaicin induces the reversible tight junctions (TJ) opening via cofilin activation. The present study investigated the mechanisms underlying the reversible TJ opening and compared the effect to the irreversible opening induced by actin inhibitors. Capsaicin treatment induced the F-actin alteration unique to capsaicin compared to actin-interacting agents such as latrunculin A, which opens TJ irreversibly. Along with TJ opening, capsaicin decreased the level of F-actin at bicellular junctions but increased it at tricellular junctions accompanied with its concentration on the apical side of the lateral membrane. No change in TJ protein localization was observed upon exposure to capsaicin, but the amount of occludin was decreased significantly. In addition, cosedimentation analyses suggested a decrease in the interactions forming TJ, thereby weakening TJ tightness. Introduction of cofilin, LIMK and occludin into the cell monolayers confirmed their contribution to the transepithelial electrical resistance decrease. Finally, exposure of monolayers to capsaicin augmented the paracellular passage of both charged and uncharged compounds, as well as of insulin, indicating that capsaicin can be employed to modulate epithelial permeability. Our results demonstrate that capsaicin induces TJ opening through a unique mechanism, and suggest that it is a new type of paracellular permeability enhancer.

## Introduction

The oral route of drug administration is considered to be the most convenient and the preferred choice for patients. However, oral administration of peptide/protein drugs encounters several obstacles. Especially, it is a great challenge to deliver hydrophilic macromolecules, as they cannot diffuse across through the lipid bilayer of the cell membrane because of their high molecular weight and hydrophilicity [1]. Therefore, enhancing the paracellular delivery of hydrophilic functional molecules including protein/peptide drugs has received considerable attention [2].

Polarized epithelial cells form tight junctions (TJ) that restrict the paracellular movement of solutes and macromolecules across epithelia. TJ are located at the apicolateral plasma membranes of adjacent cells [3] and are composed of a complex combination of transmembrane integral proteins including occludin, claudins and tricellulin, along with several intracellular proteins such as zonula occludens-1 (Zo-1), which connects the transmembrane proteins to the actin cytoskeleton [4]. Therefore, TJ proteins and the cytoskeleton are key regulators of the TJ, and TJ proteins are connected to a belt-like cytoskeleton structure to form the structural support for the TJ [5].

As noted above, the precise intracellular processes that regulate epithelial TJ permeability are of potential physiological and pharmacological significance. The controlled opening of TJ is a way to increase the absorption of hydrophilic drugs across the epithelium. In particular, reversible TJ opening could allow such drugs to be safely and controllably absorbed. This approach is attractive because it could be applied to many different hydrophilic drugs. To develop safe and effective paracellular permeability enhancers (PPEs) and improve drug bioavailability, it is necessary to understand the physiological mechanisms that regulate the structure and function of the TJ as well as paracellular permeability.

Capsaicin is the pungent ingredient in hot chili peppers. We have previously shown that capsaicin induces reversible TJ opening associated with dephosphorylation/activation of cofilin and reorganization of actin in intestinal Caco-2 cells [6]–[8]; however, the relationship between actin reorganization and reversible TJ opening remains unclear. The aim of the present study was to investigate the effect of capsaicin on the integrity of epithelial TJ at the molecular level and to analyze the mechanistic difference between reversible and irreversible TJ opening. Here we used Mandin Darby Canine Kidney (MDCK) cell monolayers, to see whether the effect of capsaicin on epithelia is general. MDCK cells are a common model for studying drug transport mechanisms [9]. The structural changes in actin cytoskeleton and epithelial junctional complex were analyzed to find differences in reversible and irreversible TJ opening, followed by cofilin and occludin overexpressions to confirm their contribution to the decrease in transepithelial electrical resistance (TER). The effect of capsaicin on permeability of several compounds was also examined and compared to known TJ modulators. The results demonstrate that capsaicin induces TJ opening through unique mechanisms and suggest that capsaicin is a new type of PPE.

## Materials and Methods

### Cell Culture and Reagents

MDCK type II cells were cultured in Dulbecco’s modified Eagle’s medium (Nacalai Tesque Inc., Kyoto, Japan) supplemented with 10% fetal calf serum (Nichirei Biosciences Inc., Tokyo, Japan) and 1% penicillin-streptomycin (Nacalai) in a humidified atmosphere containing 5% CO_2_.

Capsaicin, CytoB and FD4 were purchased from Sigma (St. Louis, MO). LatA and human recombinant insulin were from Wako Pure Chemical Industries, Ltd. (Osaka, Japan). Jpk and TFP were from Alexis/Enzo Life Sciences Inc. (Farmingdale, NY). CF was from Acros Organics (Morris Plains, NJ). Dithiobis(succinimidyl propionate) was purchased from Thermo Scientific (Rockford, IL).

Antibodies to claudin-1 (#71–7800), occludin (#71–1500), tricellulin (#700191), Zo-1 (#61–7300), E-cadherin (#33–4000), Alexa Fluor 488 goat anti-rabbit (#A11034)/anti-mouse (#A11001) IgG, and Alexa Fluor 568 goat anti-mouse IgG (#A11004) were purchased from Invitrogen (Grand Island, NY). Anti-cofilin (#3312) and anti-LIMK (#3842) were from Cell Signaling Technology (Beverly, MA). Anti-β-actin (#125K4769), anti-phospho-cofilin (Ser 3, #sc-21867-R), and β-catenin (#51–9001921) were from Sigma, Santa Cruz Biotechnology (Santa Cruz, CA) and BD Biosciences Pharmingen (San Diego, CA), respectively. Horseradish peroxidase-conjugated anti-mouse and anti-rabbit IgGs were from Kirkegaard & Perry Laboratories Inc. (Gaithersburg, MD).

All other reagents were of reagent grade and purchased from Nacalai Tesque unless otherwise noted.

### TER Measurements

For the TER experiments, MDCK cells were seeded in 12 mm-diameter transwells (pore size 0.4 µm, Corning Inc., Corning, NY) coated with collagen at a density of 1.1×10^5^ cells per well. The cells were cultured for 3 days to establish monolayer integrity. TER was measured as previously described [8].

### Immunoblotting

MDCK cells were cultured for 3 days to establish monolayers and treated with ethanol control or capsaicin solution by adding 1% volume into the medium. After specific treatments, monolayers were washed once with PBS and lysed with lysis buffer (50 mM Hepes (pH 7.4), 1% Nonidet P-40, 10% glycerol, 1 mM EDTA, 1 mM DTT, phosphatase inhibitor cocktail and protease inhibitor cocktail). After a brief sonication, the resulting cell extracts were centrifuged at 35,000×*g* for 15 min at 4°C. Supernatants with equal amounts of protein were separated by SDS-PAGE, transferred to a polyvinylidene fluoride microporous membrane (Millipore, Billerica, MA), blocked with 5% skimmed milk (Megmilk Snowbrand Co., Ltd., Sapporo, Japan), probed with the appropriate primary antibody and horseradish peroxidase-conjugated anti-IgG secondary antibody, and detected by enhanced chemiluminescence.

To detect changes in cellular F- or G-actin content, total cell extracts, cytoplasmic and cytoskeletal fractionations were prepared [10]. To obtain the membrane and cytosolic fractions, monolayers were homogenized as previously described [11].

### Fluorescence Staining and Analyses

After specific treatments, MDCK monolayers grown on LAB-TEK chamber Permanox slides (Nunc, Rochester, NY) were washed with PBS, fixed for 10 min with 3.7% formaldehyde, and permeabilized for 5 min in PBS containing 0.2% Triton X-100. After blocking with in PBS containing 5% skimmed milk for 30 min at 37°C, each antibody solution was applied and incubated for 1 h at 37°C. After washing with PBS, samples were stained with secondary antibodies as above. F-actin was labeled for 1 h with 70 nM rhodamine-phalloidin (Cytoskeleton, Denver, CO) diluted in PBS containing 5% skimmed milk and washed with PBS. The coverslips were mounted with 5 µg/ml Hoechst 33258 (Sigma) in PBS containing 60% glycerol. Fluorescence images were acquired with a Leica AF6000 deconvolution microscope system equipped with a fully automated microscope (DMI6000B) and a DFC350 FX digital camera (Leica Microsystems, Heidelberg, Germany). The *z*-stacks were collected at 0.2 µm intervals. Image stacks were deconvoluted using Leica LAS AF6000 software to generate maximum projection or 3D projection images. The fluorescence intensity of F-actin staining was measured using the software.

### Cosedimentation Studies

Velocity gradient centrifugation was performed based on a method previously described [12]. PBS containing 100 µg/ml dithiobis(succinimidyl propionate) was added to monolayers after treatment. After 5 min at room temperature, monolayers were washed five times with quenching buffer (10 mM Tris-HCl (pH 7.5), 50 mM NH_4_Cl, 120 mM NaCl), followed by lysis in 20 mM Tris-HCl (pH 7.5), 2 mM EDTA, 10 mM EGTA, 0.4% sodium fluoride and protease inhibitor cocktail at 0°C for 10 min, and centrifuged at 14,000×*g* for 30 min at 4°C. Resulting precipitates were dissolved in 0.5% Nonidet P-40, 0.25% Triton X-100, 10 mM Tris-HCl (pH 7.6), 150 mM NaCl, 1 mM EDTA, 50 mM sodium fluoride and 1 mM sodium vanadate with phosphatase/protease inhibitor cocktail at 0°C for 15 min, and centrifuged at 35,000×*g* for 15 min at 4°C. Each lysate was applied to the top of a discontinuous sucrose gradient (5, 10, 15, 20, 25%). After centrifugation at 40,000 rpm (200,000×*g*) for 18 h at 4°C with a Beckman Coulter SW 41 Ti rotor, fractions were collected from the top of the gradient. Fractions from each treatment were blotted at the same time to compare protein levels.

### Stable Transfection

A GFP-tagged human β-actin sequence was introduced into the KpnI/NotI site of the pEF1 vector (Invitrogen). Flag-HA-tagged human cofilin and LIMK sequences were cloned into the BamHI/XbaI site of the pcDNA3 vector (Invitrogen). The claudin-1 and occludin sequences were cloned into the KpnI/BamHI or KpnI/SmaI sites of pEGFP-C1 (Invitrogen), respectively. Transfections were performed using Lipofectamine LTX (Invitrogen) according to the manufacturer’s instructions. The isolation of transfectants was performed as described previously [6].

### Transport Studies

For the transport studies, MDCK cells were seeded in 6.5 mm-diameter transwells (pore size: 0.4 µm, area: 0.33 cm^2^) coated with collagen, at a density of 3.4×10^4^ cells per well. The cells were cultured for 3 days to establish monolayer integrity. TER was measured prior to each experiment to ensure the confluence of the monolayers and also during transport studies to determine the effects of the transport enhancers. Transwell plates were washed three times, incubated with Hank’s balanced salt solution (HBSS) and equilibrated for 1 h at 37°C. HBSS (0.1 ml) containing 30 µg/ml, 1.0% w/v and 0.5 mg/ml of CF, FD4 and insulin, respectively, was placed on the apical side and each transport enhancer was added to the apical side. The basolateral side was exposed to HBSS (0.6 ml), which was refreshed at predetermined intervals. Samples collected from the basolateral compartments were analyzed for CF and FD4 using a PowerscanHT fluorescence microplate reader (Dainippon Sumitomo Pharma Co., Ltd., Osaka, Japan) at an excitation wavelength of 485 nm and an emission wavelength of 530 nm. The insulin concentration was determined using an enzyme immunoassay kit (YKO 60 Human Insulin EIA kit, Yanaihara Institute, Shizuoka, Japan) according to the manufacturer’s instructions.

### Data Analysis

P_app_ was calculated according to the equation P_app_ = (*dQ*/*dt*)*(1/*A*)*(1/*C_0_*), where *dQ*/*dt* is the slope of the regression line describing cumulative amount versus time (µg/sec), *A* is the surface area of the monolayer (cm^2^), and *C_0_* is the initial penetrant concentration (µg/ml). The early phases of the measurements were used for the curve fits with the regression coefficient (r^2^) to be greater than 0.9. Statistical analyses were performed using the unpaired Student’s *t* test. Analysis of variance (ANOVA) was performed with Prism6 (GraphPad software).

## Results

### Capsaicin Induces a Reversible Decrease in TER in MDCK Monolayers

**Figure 1:**
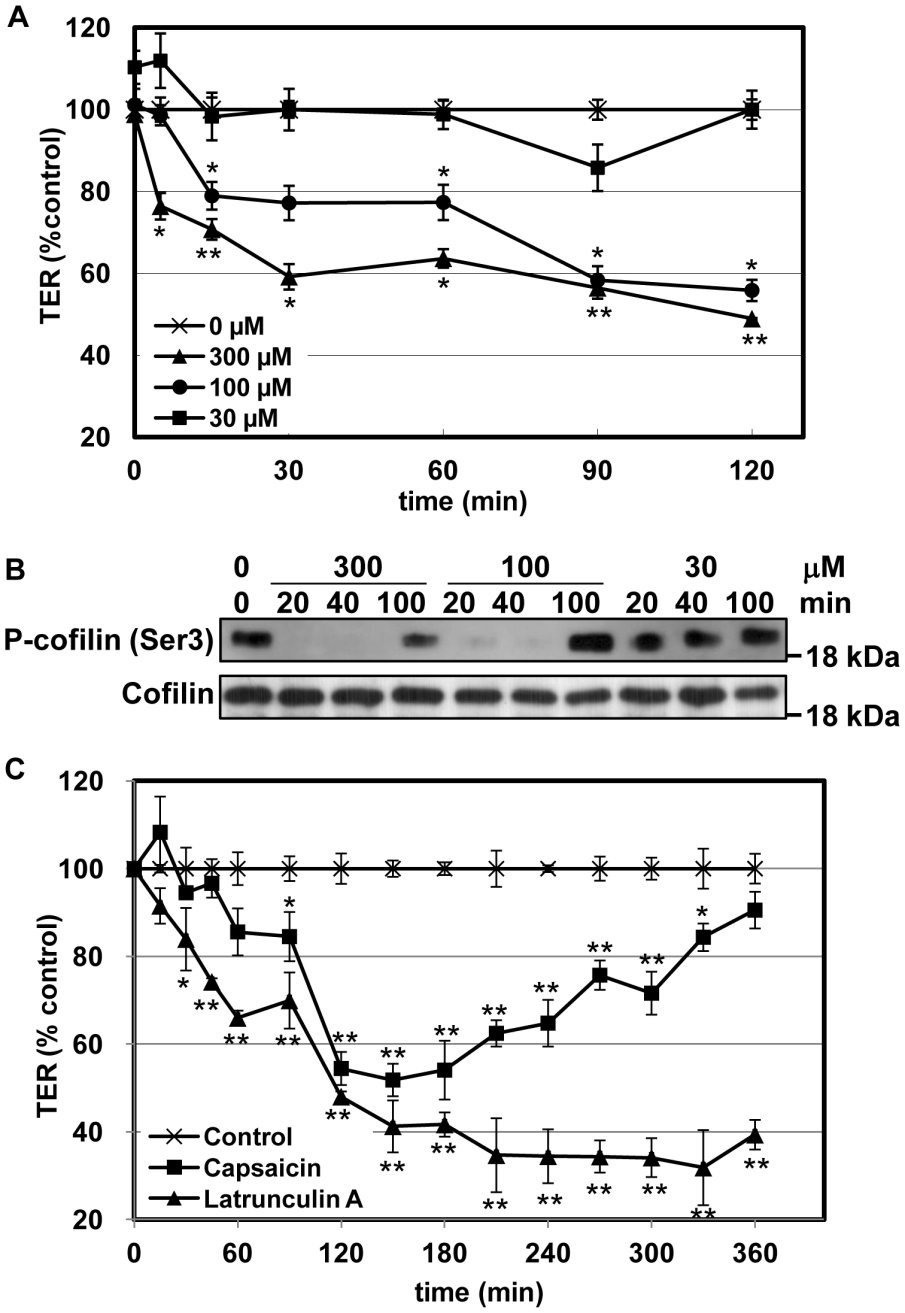
Capsaicin alters the TER of epithelial monolayers in a concentration-dependent and reversible manner. (A) TER was assessed in MDCK monolayers exposed to ethanol (vehicle) control (crosses), 300 µM (triangles), 100 µM (circles) or 30 µM (squares) capsaicin. (B) After exposure to capsaicin at the concentrations and for the lengths of time indicated, monolayers in (A) were lysed and subjected to western blot analysis. (C) MDCK monolayers incubated with ethanol control (crosses), 300 µM capsaicin (squares) or 0.1 µM LatA (triangles). Values represent mean ± S.D. Asterisks indicated significant difference from control at the same time point; *, p<0.01; **, p<0.001.

We previously showed that capsaicin decreases the TER in human intestinal Caco-2 cell monolayers. To investigate whether capsaicin is capable of opening TJ in other types of cells, MDCK monolayers were used and the degree of tightness of the TJ was measured by assessing the TER. Figure 1A shows how the addition of non-toxic concentrations of capsaicin affected the TER of MDCK monolayers. The TER was almost unchanged by treatment with 30 µM capsaicin, while 100 and 300 µM capsaicin significantly reduced the TER of the monolayers. Since we showed that capsaicin dephosphorylates/activates cofilin in Caco-2 cells as it decreases the TER [6],[7], the MDCK monolayers after the TER measurements were lysed and the level phosphorylation of cofilin was analyzed. Cofilin dephosphorylation was observed 20 and 40 min after the addition of 300 and 100 µM capsaicin, respectively, whereas cofilin was not dephosphorylated following treatment with 30 µM capsaicin (Figure 1B). This indicates that capsaicin reduces the TER and cofilin activation in a concentration-dependent manner.

Capsaicin reversibly reduces the TER in Caco-2 cells [8]. This is also the case in MDCK cells: the effect of capsaicin on the TER is reversed within several hours without removing capsaicin (Figure 1C). Importantly, cofilin dephosphorylation is also reversed after 100 min (Figure 1B), suggesting that the decrease in TER and the dephosphorylation of cofilin are correlated not only in Caco-2 cells but also in MDCK cells. Since cofilin mediates actin severing/depolymerization, it is possible that capsaicin may disrupt the actin network via cofilin activation, and that actin inhibitors may also decrease the TER. Therefore, the effects of the actin-depolymerizing agent latrunculin A (LatA) and capsaicin on the TER were compared. LatA also decreased the TER [13], but this was not reversible (Figure 1C). Because LatA irreversibly disrupts the actin network, these results suggest that reversible actin reorganization is required for reversible TJ opening.

### Involvement of a Unique Actin Alteration in Capsaicin-mediated Changes in TER

**Figure 2:**
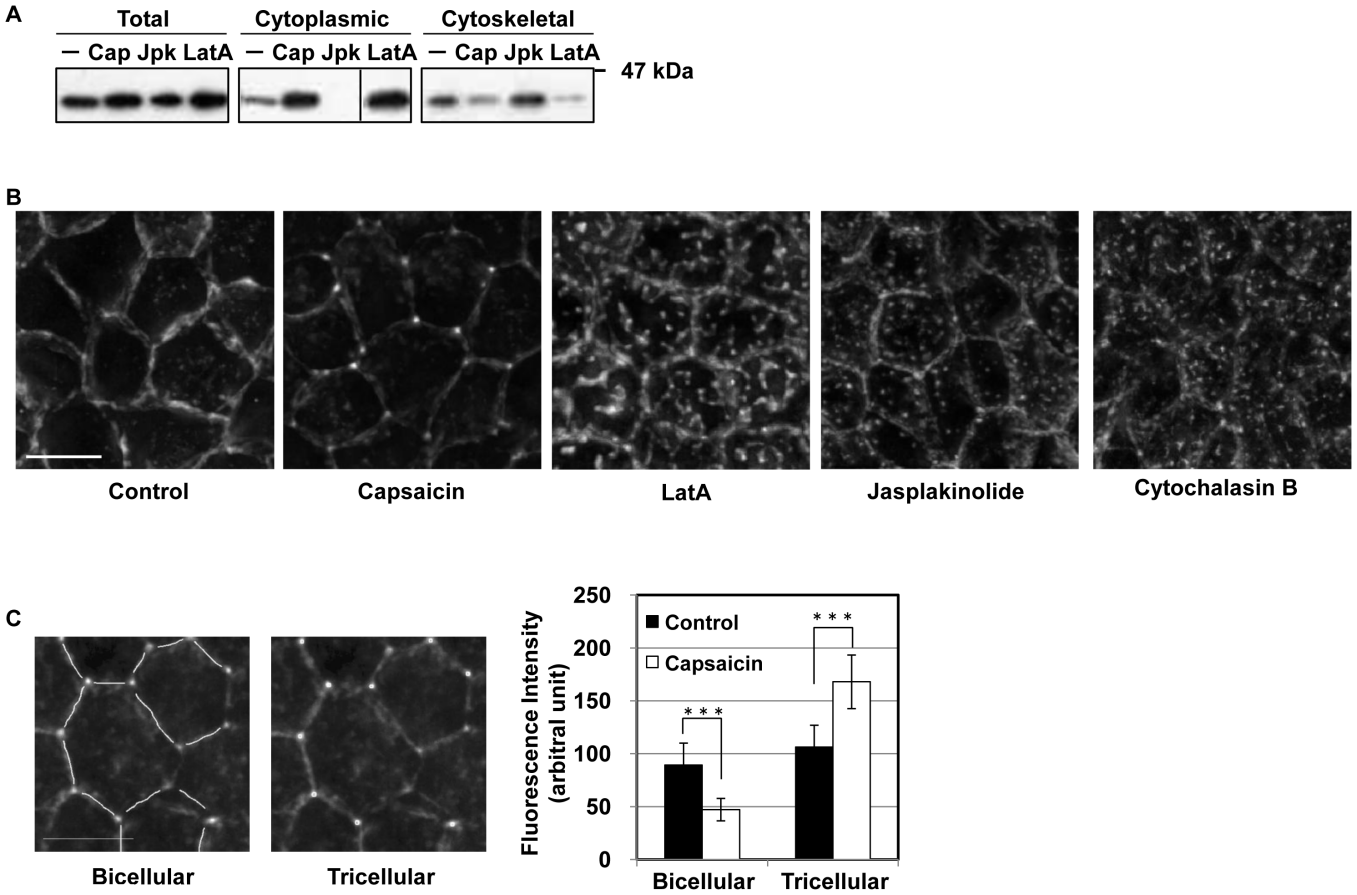
Capsaicin induces actin depolymerization and morphological changes. (A) Monolayers were exposed to ethanol control, capsaicin (300 µM, 45 min), Jpk (2 µM, 60 min) or LatA (0.5 µM, 90 min) and lysed, and the G- and F-actin fractions were separated. Fractions were analyzed by SDS-PAGE and immunoblotting with an anti-actin antibody. (B) MDCK monolayers were exposed to ethanol control, capsaicin (300 µM, 45 min), LatA (0.1 µM, 30 min), Jpk (2 µM, 1 min) or CytoB (5 µg/ml, 15 min), and were fixed and stained with rhodamine-phalloidin to detect F-actin. Images from each z-section were deconvoluted and overlayed. Bar: 10 µm. (C) The fluorescence intensities of the images in (B) were analyzed. Bar: 10 µm. ***represents p<0.001 by Student’s *t* test.

The experiments confirming the activation of cofilin by capsaicin suggest that actin disruption is involved in TJ dysfunction. Actin depolymerization decreases the TER [13]–[15]; however, our data show that capsaicin, but not LatA, decreases the TER reversibly, suggesting that the two agents engage different mechanisms. Therefore, the effects of the two agents on actin were compared to identify the capsaicin-specific mechanism of reversible TJ opening. The effect of capsaicin on G- and F-actin distribution was investigated first. Treatment with capsaicin increased the G-actin and reduced the F-actin content after 45 min, while the actin-stabilizing agent jasplakinolide (Jpk) had the opposite effect (Figure 2A). LatA induced the same distribution changes as capsaicin. This supports the notion that capsaicin induces cofilin activation and modulates the state of actin from filamentous to globular.

Next, to identify morphological changes in perijunctional actin structure that may correlate with the loss of barrier function and specifically occur following capsaicin treatment, monolayers were fixed and stained with rhodamine-phalloidin. Morphological changes were detected approximately 15 min after the addition of capsaicin, almost at the same time as the decrease in TER. Capsaicin induced morphological changes that reduced the level of perijunctional actin at bicellular junctions and produced actin-rich regions at tricellular junctions (Figure 2B). LatA, cytochalasin B (CytoB) and Jpk induced the formation of randomly dispersed actin aggregates.

Next, GFP-actin stable MDCK transfectants were established for live cell imaging to observe changes in actin morphology in detail. Time-lapse imaging allows the precise monitoring of capsaicin-specific actin changes (Movie S1 in the supporting information). Capsaicin increased the concentration of GFP-actin in a small zone at trijunctional TJ (triTJ) with reduced fluorescence at bijuntional TJ (biTJ). To quantify this difference, the intensity of fluorescence was measured separately at biTJ and triTJ. The decrease in actin intensity at biTJ and the increase at triTJ were significant and confirmed the morphological changes mentioned above (Figure 2C).

Taken together, capsaicin induces actin depolymerization and thereby increases the level of G-actin, which is consistent with cofilin activation. However, unlike direct actin-depolymerizing/stabilizing agents, which separate actin randomly to produce many small aggregates, capsaicin-induced actin depolymerization mainly occurs at biTJ, suggesting that F-actin persists at triTJ. To our knowledge, this actin alteration is capsaicin-specific.

### Capsaicin Affects the Subcellular Lateral Localization of F-actin Relative to TJ Proteins

**Figure 3:**
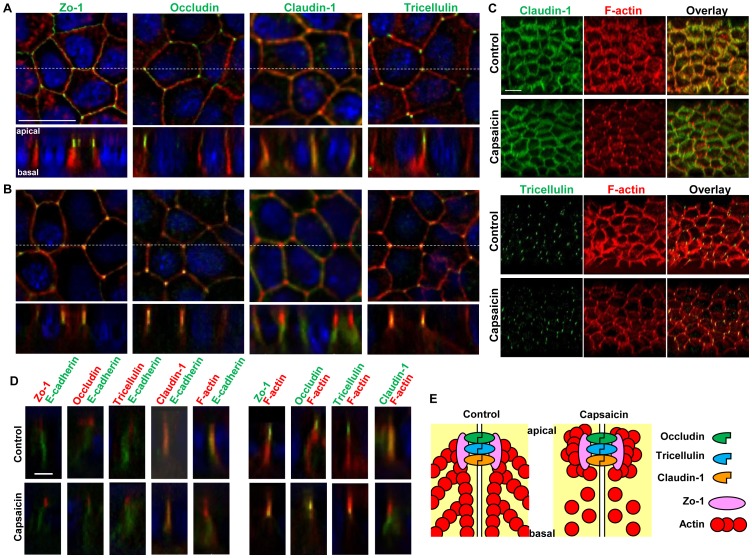
Effects of capsaicin on the distribution of TJ proteins and F-actin. (A, B) Monolayers were exposed to vehicle (A) or 300 µM capsaicin (B) for 45 min and were labeled with each TJ antibody (green), rhodamine-phalloidin (red) and Hoechst (blue). Images were collected as a Z-series, and then deconvoluted and overlayed to display a single composite projection. *Top*: XY sections of merged images. *Bottom*: XZ sections of merged images. Scale bar: 10 µm. The corresponding TJ antibodies are listed above the images. (C) 3D projection images in a 45°-angle of claudin-1 and tricellulin staining, plus F-actin. Scale bar: 10 µm. (D) MDCK monolayers exposed to vehicle (top) or capsaicin (bottom) as above were stained with E-cadherin. The colors corresponding to each antibody are listed above the images. Scale bar: 2.5 µm. (E) Schematic explanation of the protein distributions.

Next, the relationship between the actin alteration described above and TJ structural components was examined. For this purpose, confluent MDCK monolayers exposed to ethanol (vehicle) control or 300 µM capsaicin for 45 min were examined by immunofluorescence microscopy. The distributions of F-actin, Zo-1, a submembranous protein known for its scaffolding characteristics, and three transmembrane proteins, claudin-1, occludin and the trijunctional protein tricellulin, were analyzed in XY and XZ sections. Figure 3A shows the ring-like structures of Zo-1, occludin and claudin-1 on the lateral membranes between neighboring cells, with tricellulin only present at triTJ in control monolayers. F-actin staining also showed perijunctional actin ring structures. The XZ sections of control cells showed the typical dotted staining of Zo-1, occludin and tricellulin at the apical tip of the lateral membrane, and claudin-1 localized along the lateral membrane and at the apical tip. This lateral membrane distribution of claudin-1 has been observed previously [16]. In capsaicin-treated monolayers (Figure 3B), F-actin concentrated to triTJ in XY sections and was closer to Zo-1, occludin and tricellulin in XZ sections. Therefore, capsaicin seems to induce the appearance of F-actin to a greater extent on the apical side than the basal side. This was continuous along the monolayers as seen in the 3-dimensional (3D) projection images (Figure 3C).

To confirm this phenomenon, the relative distributions of the proteins mentioned above and E-cadherin, an adherens junction protein, were analyzed in Z-sections. In control monolayers, Zo-1, occludin and tricellulin were localized to the apical side of the membrane relative to E-cadherin, and F-actin and claudin-1 were observed almost at the same depth (Figure 3D). This is consistent with the relative distribution observed in Figure 3A and B. In capsaicin-treated monolayers, the relative distribution of TJ proteins compared to E-cadherin was unchanged; however, F-actin was shifted above E-cadherin, increasing the colocalization area with Zo-1, occludin and tricellulin. Taken together, this analysis suggests that capsaicin induces a shift in F-actin to the apical side relative to TJ proteins, with no significant changes in TJ protein localization (Figure 3E). To our knowledge, this observed pattern is also capsaicin-specific in contrast to LatA and Jpk that affect TJ structure/organization [13],[17].

### Occludin Protein Content and the Protein-protein Interactions Involving Occludin Reduced in TJ

**Figure 4:**
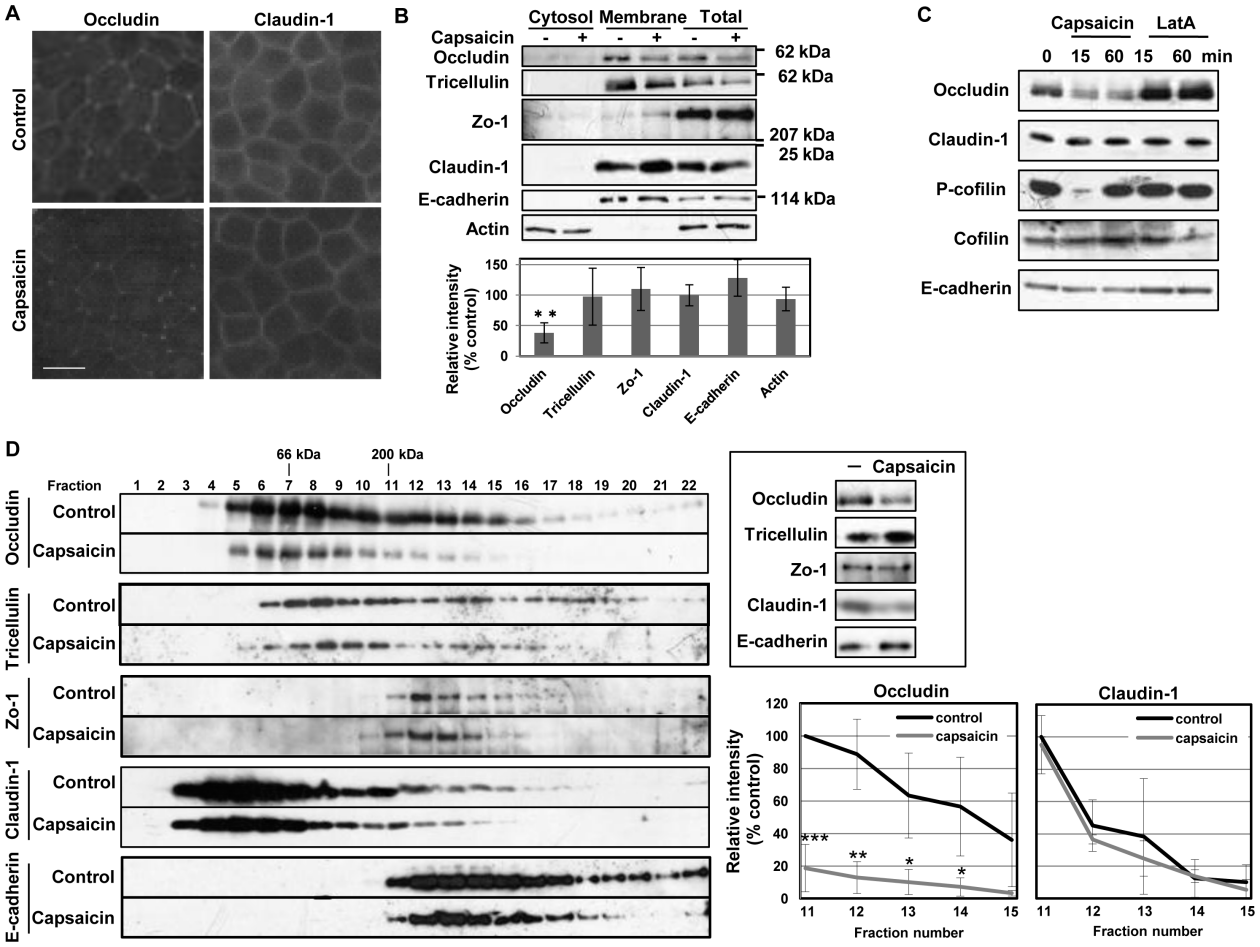
Capsaicin decreases occludin protein content and the protein-protein interactions involving occludin in TJ. (A,B) MDCK monolayers were exposed to ethanol control or 300 µM capsaicin for 45 min. (A) The cellular localization of the TJ proteins occludin and claudin-1 was examined by immunofluorescence. Scale bar: 10 µm. (B) Western blot detection of occludin, tricellulin, Zo-1, claudin-1, E-cadherin and actin in the cytosol, membrane fractions and total cell extracts. The densitometic analysis of total proteins from three independent experiments performed with NIH ImageJ software. **represents p<0.01 by Student’s *t* test. (C) Western blot detection of occludin, claudin-1, phospho-cofilin, cofilin and E-cadherin in total extracts exposed to 300 µM capsaicin or 0.1 µM LatA for the durations indicated. (D) Distribution of TJ proteins in sucrose gradient centrifugation of lysates from monolayers (shown in the inset) after exposure to ethanol control or 300 µM capsaicin for 1 h. Fractions were collected from the top of the gradient, separated by SDS-PAGE and subjected to immunoblotting. The apparent molecular masses of BSA (66 kDa) and myosin (200 kDa) are shown above the fraction number. Representative data from three independent experiments is shown. The densitometic analyses from three independent experiments are shown. *, p<0.05; **, p<0.01; ***, p<0.001.

During the analysis of TJ components localization, we noticed the staining intensity of occludin diminished in capsaicin-treated monolayers. In fact, the intensity of occludin was markedly decreased compared to that of claudin-1 unchanged (Figure 4A), suggesting that capsaicin may decrease the levels of occludin. Therefore, the levels of TJ proteins were assessed by western blotting of monolayers with or without capsaicin treatment. The cytosolic and membrane fractions were analyzed separately because some TJ-altering treatments displace TJ proteins into the cytosol [11],[13]. In non-treated cells, occludin, tricellulin, claudin-1 and E-cadherin were mainly distributed in membrane fractions, and Zo-1 appeared in both fractions (Figure 4B). Capsaicin decreased the level of occludin in total cell extracts and membrane fractions without increasing the level in the cytosolic fraction, whereas the levels and distributions of claudin-1 and E-cadherin were unchanged.

To determine whether the decreased occludin level and cofilin activation were capsaicin-specific phenomena, the effect of LatA, which decreases the TER irreversibly, on occludin and cofilin was assessed. Compared to capsaicin, LatA (0.1 µM) considerably decreased the TER at 60 min (Figure 1C); however, occludin expression and cofilin phosphorylation were not affected (Figure 4C). These data indicate that the decrease in occludin level and actin reorganization mediated by cofilin activation are capsaicin-specific mechanisms that reversibly increase TJ permeability.

To assess the physiological/physical meaning of the decrease in the level of occludin, cosedimentation analysis was performed to investigate whether this decrease affects protein-protein interactions in TJ complexes. When monolayer lysates were subjected to velocity gradient centrifugation on sucrose gradients, part of occludin cosedimented with Zo-1, claudin-1 and tricellulin in fractions 11–15 (Figure 4D), which is similar to the cosedimentation pattern of TJ protein complexes previously described [12]. Their apparent molecular mass is greater than 200 kDa (Figure S1A in the supporting information), which is higher than the monomeric mass of each. To confirm that these are not monomeric proteins, the monolayers were lysed with 1% SDS; occludin and Zo-1 concentrated in smaller molecular weight fractions (fractions 5 to 10, Figure S1B in the supporting information) as monomers [18]. Taken together, figure 4D shows that the level of occludin, but not Zo-1 or claudin-1, is decreased in fractions 11–15, which contain TJ protein complexes following capsaicin treatment. These results suggest that the decrease in the level of occludin observed in total lysates following capsaicin treatment reflects a decrease in occludin complex formation within TJ.

### Relationship between the Signaling Pathways Involved in the Capsaicin-dependent Activation of Cofilin and the Decreased Level of Occludin

The analysis described above suggests that capsaicin modulates two major molecules in TJ structures: capsaicin decreases and changes the F-actin distribution, and decreases the level of occludin in TJ. Therefore, unlike the actin-depolymerizing factors that induce TJ opening through the loss of actin network, capsaicin seems to increase TJ permeability by modulating not only actin but also TJ structure, i.e., by altering the levels and interactions of the proteins constituting the TJ. Therefore, the relationship between these two signaling pathways was studied, and the contribution of F-actin and occludin to the capsaicin-induced decrease in TER was assessed.

**Figure 5:**
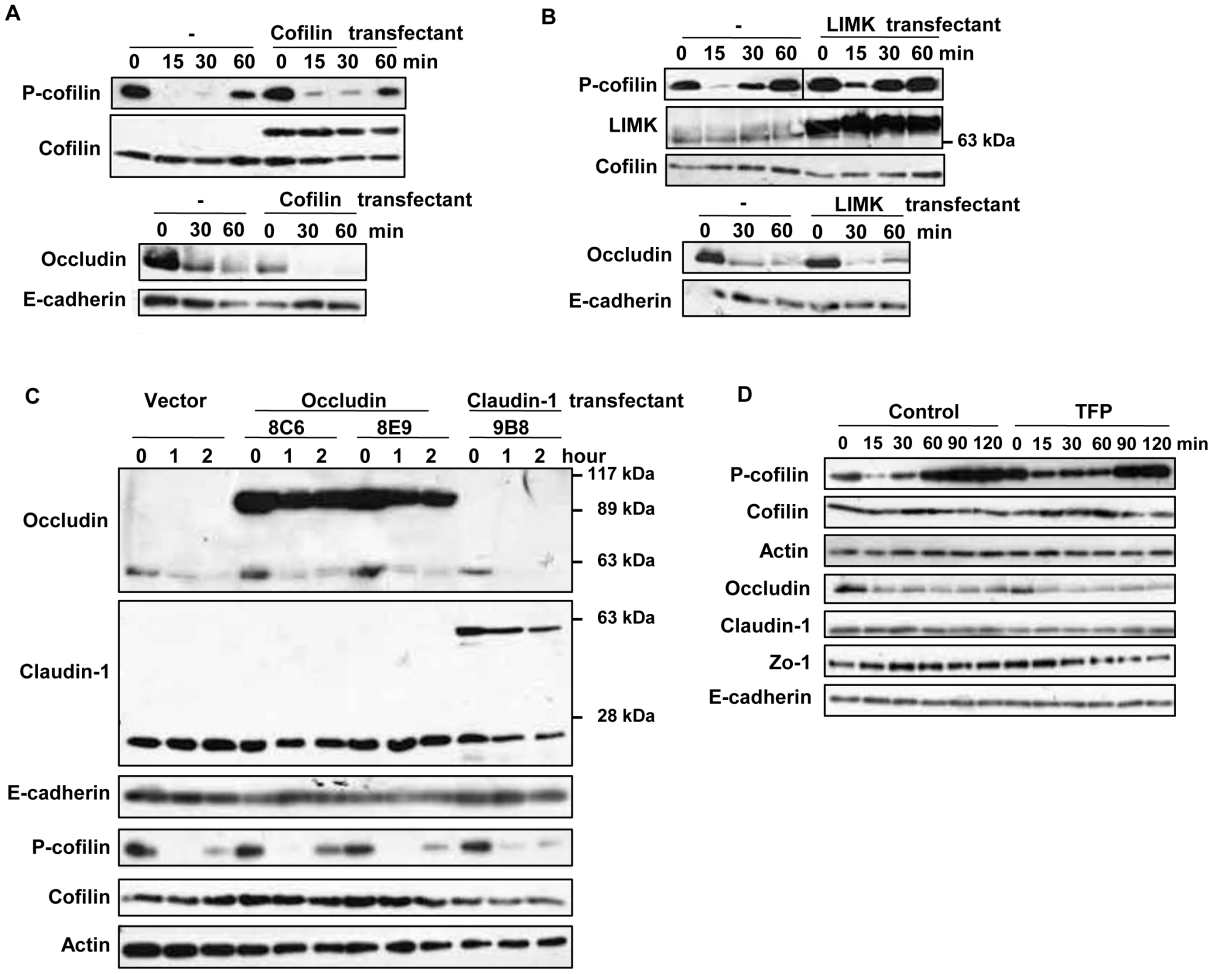
Effects of suppression of cofilin activation or overexpresssion of TJ proteins on the capsaicin-induced cofilin dephosphorylation and decrease in occludin expression. (A–C) Flag-HA tagged cofilin and LIMK, and EGFP-occludin and claudin-1 stable transfectants were established. Monolayers prepared from the transfectants and non-transfected MDCK cells were exposed to 300 µM capsaicin for the time indicated. After lysis, protein expression was analyzed by immunoblotting. Each experiment was performed with at least two different clones and repeated at least twice. (D) MDCK monolayers were pretreated with vehicle or 50 µM TFP for 30 min, and then exposed to 100 µM capsaicin for the time indicated. The levels of occludin and phospho-cofilin were analyzed by western blotting.

First, the effect of cofilin activation on the decrease in the level of occludin was analyzed, and vice versa. Cofilin is expected to be the upstream molecule in the capsaicin-induced F-actin reorganization [19]. LIMK (LIM kinase), which phosphorylates and inactivates cofilin, and cofilin were introduced into MDCK cells, and stable transfectants were established. In the cofilin stable transfectants, cofilin dephosphorylation induced by capsaicin was reduced (Figure 5A). The same was observed in LIMK transfectants (Figure 5B). However, the capsaicin-induced decrease in occludin was not changed. Next, occludin and claudin-1 were stably expressed in MDCK monolayers with fluorescent protein tags [13]. The distribution of these fusion proteins was assessed by microscopic analysis: the tagged proteins showed the ring-like structures as their endogenous counterparts (Figure S2 in the supporting information). Since stable occludin expression produced one clone (8C6) with similar TER as non-transfected MDCK cells and other clones with a ∼30% increase in TER (claudin-1 and LIMK expression also increased TER), two clones are shown for occludin. Claudin-1 expression had no effect on the decrease in occludin or cofilin dephosphorylation induced by capsaicin. Following the overexpression of occludin, the decrease in endogenous occludin induced by capsaicin was not inhibited, but exogenous EGFP-occludin overwhelmed the decrease; however, cofilin dephosphorylation was not affected. In addition, trifluoperazine (TFP) inhibited capsaicin-induced cofilin dephosphorylation by blocking calcium signaling [6],[7], but the occludin decrease was not affected (Figure 5D). These observations suggest that the capsaicin-induced cofilin dephosphorylation and decrease in the level of occludin are independent events.

### Cofilin Activation and a Decreased Level of Occludin Contribute to the TER Decrease Induced by Capsaicin

**Figure 6:**
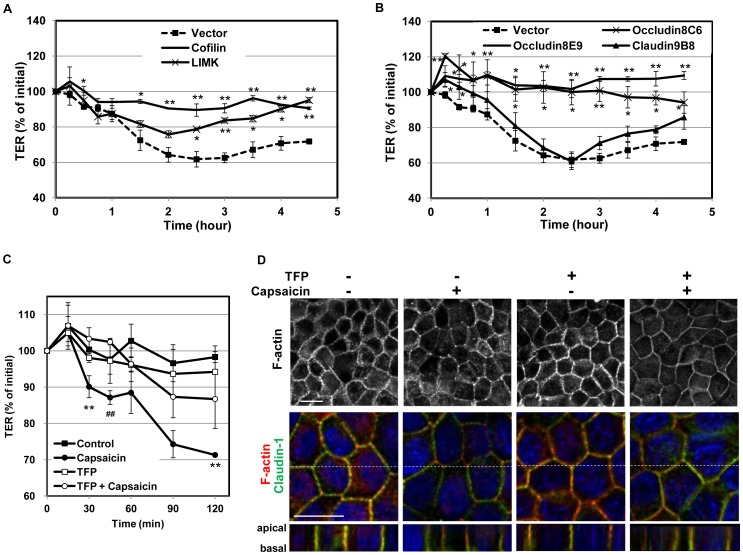
Overexpresssion of cofilin, LIMK and occludin, but not claudin-1, significantly diminishes the capsaicin-induced decrease in TER. (A,B) Transfectant monolayers were prepared in transwell inserts, exposed to 300 µM capsaicin, and subjected to TER measurements. Values represent mean ± S.D. Asterisks indicated significant difference from Vector control at the same time point; *, p<0.01; **, p<0.001. (C) TER was assessed in MDCK monolayers pretreated with vehicle control or 50 µM TFP for 30 min, and exposed to 100 µM capsaicin at time 0. Values represent mean ± S.D. Repeated measure ANOVA followed by Tukey’s multiple comparisons test; **p<0.01, control vs. capsaicin; ^##^p<0.01, TFP + capsaicin vs. capsaicin. Each experiment was performed with at least two different clones and repeated at least twice. (D) MDCK monolayers were treated with vehicle or 50 µM TFP for 30 min followed by 100 µM capsaicin for 15 min, then fixed and stained with rhodamine-phalloidin to detect F-actin alteration (upper panel). To see the relative distribution of F-actin (red) in 3D, images labeled with anti-claudin-1 (green) and Hoechst (blue) are also shown below. Images from each z-section were deconvoluted and overlayed. Bar: 10 µm.

Next, the effect of each of these two independent phenomena on the TER decrease was investigated. Vector control, cofilin and LIMK stable transfectants formed monolayers on transwells and were exposed to capsaicin. The decrease in TER was significantly reduced in the cofilin and LIMK transfectants compared to that of vector transfectants, suggesting that cofilin dephosphorylation is required for capsaicin-induced TJ opening (Figure 6A). Next, the decrease in TER was examined in EGFP-occludin and EGFP-claudin-1 transfectants exposed to capsaicin. Claudin-1 transfectants displayed a similar decrease in TER to vector transfectants; however, occludin transfectants showed almost no decrease, suggesting that efficient occludin degradation is also required for capsaicin-induced TJ opening (Figure 6B). As we observed that the capsaicin-induced cofilin dephosphorylation but not the decrease in the level of occludin was inhibited by TFP in Figure 5D, we also examined whether TFP treatment affects the TER decrease. Figure 6C demonstrated that TFP co-treatment with capsaicin significantly inhibited the decrease in TER. In this condition, capsaicin-specific actin alteration was also inhibited (Figure 6D), correlating the level of cofilin phosphorylation. Taken together, these observations suggest that cofilin activation and a decreased level of occludin are independent of one another but that both of them contribute significantly to the decrease in TER induced by capsaicin. The actin reorganizatino is correlated to the level of cofilin activation, suggesting that the cofilin-actin modulation is one of the pathways required for the TJ opening by capsaicin.

### The Actin Alteration Induced by Cofilin Correlates with the Reversibility of TJ Opening

**Figure 7:**
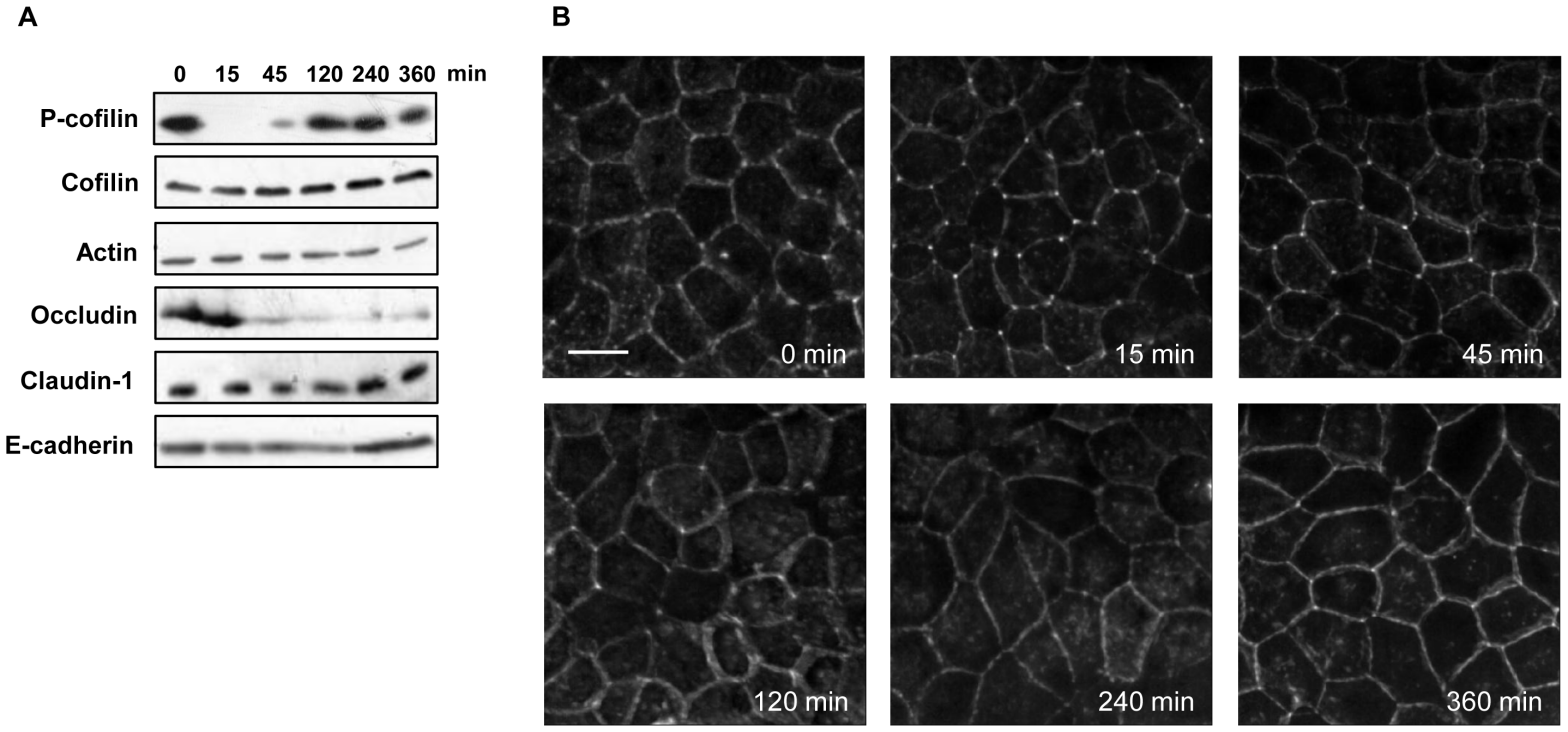
Cofilin activation, decrease in occludin expression and actin alteration in the recovery phase of capsaicin treatment. (A) Western blot detection of phospho-cofilin and occludin in total extracts exposed to 300 µM capsaicin for the times indicated. (B) MDCK monolayers were exposed to 300 µM capsaicin for the time indicated, and then fixed and stained with rhodamine-phalloidin to detect F-actin. Images from each z-section were deconvoluted and overlayed. Bar: 10 µm.

With the understanding that both cofilin dephosphorylation and a decrease in the level of occludin are necessary for efficient TJ opening, the contribution of each to the reversibility of TJ opening was investigated. For this purpose, cofilin dephosphorylation and the decrease in the level of occludin were analyzed for a longer period of time. Cofilin dephosphorylation was induced as early as 15 min after the addition of capsaicin and started to recover after 45 min (Figure 7A). At 120 min, cofilin was phosphorylated almost to the same extent as before treatment. By contrast, the occludin decrease was significant throughout the entire time course from 45 to 360 min. Since the TER was decreased until 120 min and then started to increase to reach full recovery at 360 min (Figure 1C), cofilin phosphorylation/inactivation, rather than the decrease in occludin, preceded the recovery.

To determine whether cofilin inactivation contributes to the recovery phase, actin alteration was analyzed by rhodamine-phalloidin staining during the same time period as in Figure 7A. After 15 and 45 min of capsaicin treatment, F-actin was concentrated at triTJ and was reduced at biTJ, which is a capsaicin-specific actin alteration as shown in Figure 2B (Figure 7B). This alteration began to disappear at 120 min, after which the distribution of F-actin looked similar to that before treatment (0 min). The timing of these changes is similar to that of cofilin activation. These observations show that the reversal of TJ opening induced by capsaicin is not dependent on the decreased level of occludin but correlates with the specific actin alterations induced by cofilin activation.

### Capsaicin Increases the Permeability of Non-ionic/Ionic Molecules in MDCK Monolayers

TJ also control the permeability of the paracellular pathway, which can be measured via the diffusion of molecules of different sizes. The effect of capsaicin on the passage of three different molecules, 5(6)-carboxyfluorescein (CF), FITC-dextran-4 (FD4), and insulin (376 Da, 4.4 kDa, and 5.8 kDa, respectively) was assessed. The molecules were applied to the apical side of the MDCK monolayers in transwells to measure permeability.

**Figure 8:**
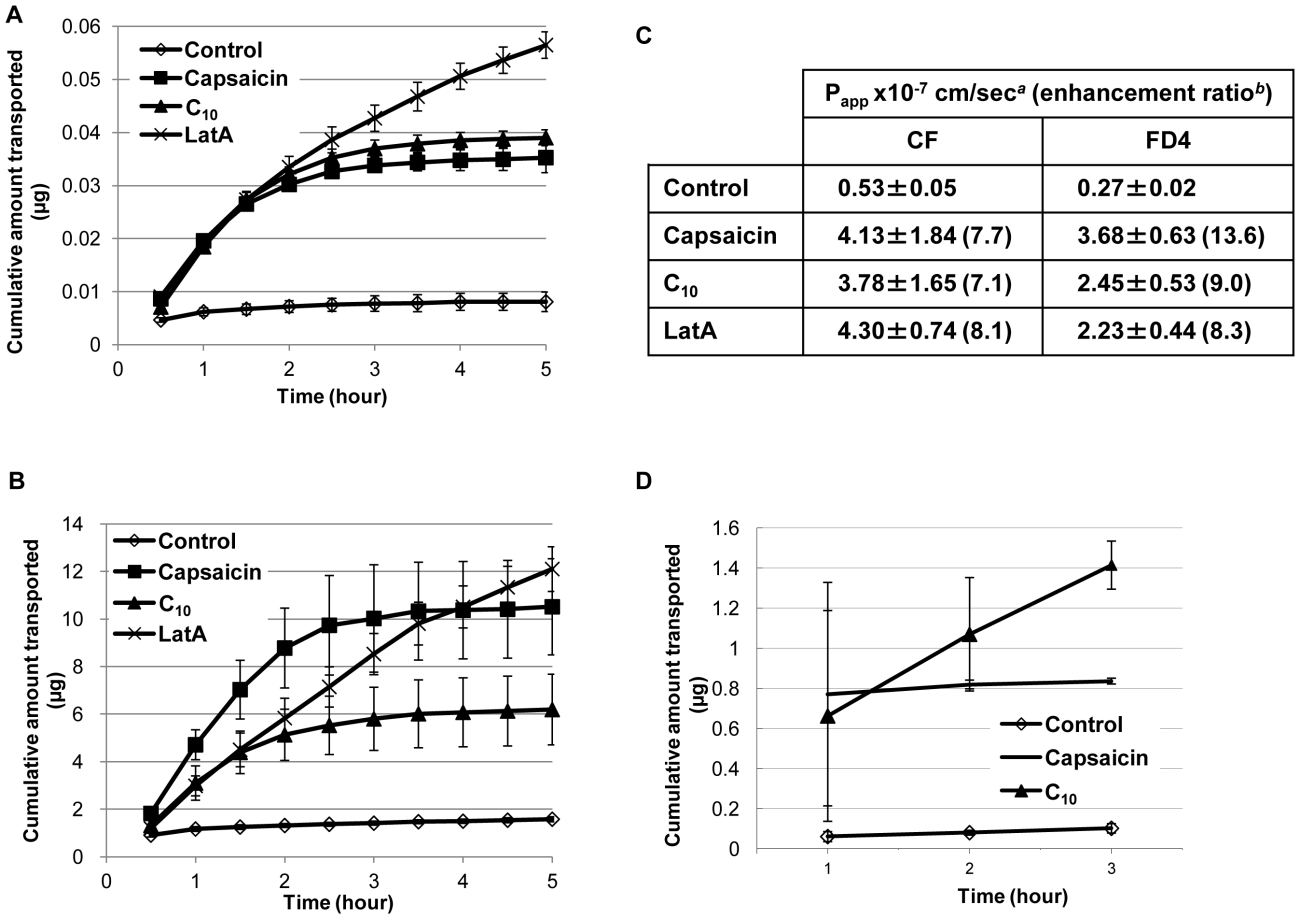
Capsaicin increases TJ permeability as measured by the paracellular passage of ionic and non-ionic compounds. (A,B,D) MDCK cells were seeded on transwells and grown for 3 days to develop monolayers. Paracellular fluxes of CF (A), FD4 (B), and insulin (D) were measured in the apical to basolateral direction. All measurements were in triplicates (n = 3) and representative data are shown. (C) Effects of 300 µM capsaicin, 1 mg/ml C_10_, 0.1 µM LatA on the P_app_ of molecules across MDCK monolayers. P_app_ was calculated from the above data. *^a^*Each value represents the mean ± S.D. of three independent experiments. *^b^*Enhancement ratio calculated as P_app_(sample)/P_app_(control).

An approved rectal absorption enhancer, sodium decanoate (C_10_) [22], significantly increased the passage of CF and FD4 (Figure 8A and B). Capsaicin increased CF permeability to a similar extent as C_10_. Capsacin increased FD4 permeability more than C_10_ did. LatA also increased the permeability, at first to a lower extent than the other two agents, and then to a larger extent in the later phase of the measurement. This is probably because the permeability enhancing effects of capsaicin reversed after several hours, while those of LatA did not. This is consistent with the TER measurement results in Figure 1C, showing that the effect of capsaicin on TER, but not of LatA, is reversible even though capsaicin is not depleted. These observations suggest that capsaicin-induced TJ permeability is temporal and recovers spontaneously. The penetration-enhancing effects of these agents were also evaluated in terms of the apparent permeability coefficient (P_app_) for each molecule (Figure 8C). The P_app_ values of capsaicin were less dependent on molecular size and capsaicin increased permeability more than C_10_.

Capsaicin also increased insulin permeability (Figure 8D). The effect of capsaicin on the passage of insulin was similar to that of C_10_ at 2 h, after which it stopped, again indicating spontaneous recovery and that it can be easily controlled. The recovery phase of capsaicin treatment affected the passage of insulin more abruptly than the passage of CF and FD4, reflecting that the passage of charged high molecular weight molecules is more sensitive to capsaicin recovery than the passage of low molecular weight or uncharged molecules.

In conclusion, the present study demonstrates that capsaicin is capable of opening the TJ of epithelial cells in a reversible and concentration-dependent manner. Capsaicin modulates TJ through at least two mechanisms: changes in the polymerization state and subcellular distribution of actin, and a decrease in the TJ occludin level. Stable transfectants showed that both actin-depolymerizing factor (cofilin) activation and a decreased level of occludin are important for the effective and significant TER decrease induced by capsaicin, although the recovery correlates with cofilin inactivation and actin assembly but not with decreased occludin. Finally, the study confirmed that capsaicin increases the paracellular permeability of both charged and uncharged compounds with spontaneous recovery, which is consistent with a reversible decrease in TER. Taken together, the identification of the effect of capsaicin on TJ and that of a new mechanism of reversible TJ opening raise the possibility of developing a novel kind of PPE.

## Discussion

In this study, capsaicin was shown to reversibly modulate the TER and paracellular permeability of MDCK monolayers within several hours, other than Caco-2. Although a few virus-derived proteins/peptides induce a TER decrease reversibly within hours [20],[21], most natural or synthetic molecules reported to induce TJ opening have an irreversible effect or an effect reversible only after removal of the compound [13],[22]–[25]. Therefore, the present study focused on the mechanisms underlying the reversible modulation of TJ by capsaicin, with the aim of applying the findings to the development of novel PPEs.

The effect of capsaicin on actin was investigated first, since we have previously reported that capsaicin induces cofilin activation and actin alteration in Caco-2 [6],[7]. Drugs that stimulate actin depolymerization and actomyosin contraction induce paracellular permeability [13],[23],[26],[27]; however, the drug must be removed to reverse these effects. Therefore, we hypothesized that capsaicin has a different mode of action on actin, and we analyzed the details and differences in actin modulation by capsaicin and LatA, which induces an irreversible decrease in TER (Figure 1C).

Because cofilin is an actin-depolymerizing factor, treatment with capsaicin increased the level of G-actin and decreased the level of F-actin, similar to the effects of LatA, an actin depolymerization agent (Figure 2A). However, treatment with capsaicin induced marked differences in F-actin structure compared to the effects of treatment with LatA, Jpk and CytoB, which interact directly with actin (Figure 2B). In addition, opposite changes at triTJ and biTJ were observed by time-lapse microscopy following capsaicin treatment: the level of actin decreased at biTJ and increased at triTJ (Movie S1 in the supporting information). This capsaicin-specific change in F-actin was confirmed (Figure 2C), suggesting that the capsaicin mode of action is quite different from that of direct actin-depolymerizing agents.

The characterization of capsaicin-specific actin reorganization is indicating that there are capsaicin-specific mechanisms responsible for reversible TJ modulation; therefore the effect of capsaicin inside TJ structure was investigated. Immunofluorescence microscopy was used to study actin within TJ structures, along with TJ proteins themselves. Control cell monolayers displayed the typical regular organization of fully differentiated MDCK cells, with F-actin localized laterally at cell-to-cell contact points in polarized cells (Figure 3A) [28]. TJ proteins appeared at the cell membrane in XY sections and in XZ sections along with lateral plasma membranes [28]–[30]. F-actin in cell-to-cell contacts was still present in cells treated with capsaicin (Figure 3B), but was concentrated apically, indicating that a cytoskeletal rearrangement occurred on the lateral side. Taken together, capsaicin modulates not only actin polymerization but also the 3D F-actin subcellular distribution. This was generally a homogeneous change in treated cells (Figure 3C), rather than an irregular disruption of F-actin [30],[31]. The localization of TJ proteins was not significantly changed by capsaicin treatment (Figure 3D), indicating that the polarized organization of TJ had not been altered, in contrast to LatA-induced perturbation [13],[17]; however, the total occludin content was decreased upon exposure to capsaicin (Figure 4A, B). Cosedimentation analysis revealed that the level of occludin was decreased in TJ protein complex fractions, indicating a decreased interaction between occludin and other TJ components (Figure 4D). Taken together, these results suggest that the actin alteration and decrease in the level of occludin induced by capsaicin affect TJ integrity, which leads to TJ opening.

Several types of proteins contribute to the maintenance of TJ [32]; among them, the F-actin cytoskeleton and occludin are important components. The binding of Zo proteins to the intracellular C-terminal tail of occludin anchors the TJ protein complex to the actin cytoskeleton [33],[34]. These interconnections are believed to stabilize the TJ and to be critical to its regulation [5]. Occludin expression levels have also been strongly linked to elevated permeability [35]–[37]. The present study found that there are two capsaicin-specific events, i.e., unique actin reorganization via cofilin and a decrease in the level of occludin, since LatA treatment did not induce any of them (Figure 4C). This suggests that one or both of these changes may be linked to reversible TJ opening.

Given the above observations, the relationship between capsaicin-induced cofilin activation and decreased occludin expression was analyzed. The two events are independent of one another, but both of them are necessary to induce a significant decrease in TER (Figure 5, 6). Inhibition of cofilin activation by cofilin/LIMK overexpression or TFP co-treatment reduces the TER loss, with induction of occludin expression decrease. Meanwhile, occludin overexpression abolishes the TER decrease without affecting cofilin activation. Therefore, cofilin must be activated and occludin expression must be decreased concomitantly to induce an efficient decrease in TER upon exposure to capsaicin. Actin alteration and decreased occludin expression can each open TJ separately if strong enough, similar to other TJ modulators, as described above. The direct actin depolymerization and discontinuous TJ structure induced by LatA might be too strong for spontaneous recovery. The combination of the indirect (via cofilin) actin depolymerization/reorganization and the decrease in occludin expression that occurs without relative localization changes in TJ structure might be important for the reversible, yet effective, permeability increase induced by capsaicin.

Our study also yields insights into recovery, i.e., the closing phase of TJ, because of the reversibility of the effect of capsaicin. Cofilin activation and actin reorganization were attenuated after approximately 120 min of capsaicin treatment, whereas the level of occludin remained low until 6 hours, that is at recovery phase (Figure 7). Therefore, actin alteration via cofilin seems to correlate with reversible TJ opening. This is consistent with the fact that LatA induces actin depolymerization irreversibly, resulting in an irreversible TER decrease.

In addition, these observations deepen the insights of occludin role in the barrier function. As we have seen here and other literatures had shown, the perturbations of occludin increased TJ permeability [35]–[37]. However, the findings about occludin-deficient cells that develop TJ and following studies demonstrated that occludin is not required for TJ barrier function [38]. That means if occludin has been lost during TJ formation, it can be dispensable or compensated. This can happen when the barrier function recovered from capsaicin treatment with persisting occludin decrease. It is also noteworthy that occludin knockdown unchanged steady-state TER [39] or just delayed TJ assembly [40], suggesting that occludin decrease affects the process of TJ assembly but still able to establish TJ barrier function. Taken together, occludin is playing an important role to regulate the permeability of TJ which already established with its existence, but it is not essential for forming TJ barrier. It is also possible that dynamics of other TJ proteins manage TJ strands to be fine-tuned when occludin decreased. Because such as MarvelD3 is able to partially compensate for occludin loss [40], and tricellulin distributes more evenly along the junction in the absence of occludin [41]. Therefore, when occludin expression is decreased continuously, other TJ strands associated proteins might compensate for the loss of occludin, again that might enable the TJ functional recovery without occludin expression recovery. Further study is underway to investigate these observations.

Finally, the present study suggests that capsaicin could be employed as a potential tool for the modulation of TJ permeability. The absorption of large and hydrophilic molecules, which include numerous therapeutic compounds, is limited by TJ, which control passage through the paracellular pathway. Capsaicin can enhance the epithelial permeability of insulin (Figure 8). Capsaicin has advantages, including the induction of reversible permeability, safety (being derived from food ingredients), and ease of co-administration with biologically active compounds.

In conclusion, the study demonstrated that capsaicin is capable of transiently opening TJ. Actin and occludin take part in reversible TJ modulation. This knowledge can be applied to the development of novel PPEs.

## Supporting Information

Figure S1 **Distributions of monomeric standards and TJ proteins.** (A) BSA (Sigma) and myosin (prepared from rabbit muscle) were dissolved in the cosedimentation lysis buffer and loaded on sucrose gradient for velocity gradient centrifugation. Fractions were analyzed by Coomassie Brilliant Blue staining. (B) A control monolayer without dithiobis(succinimidyl propionate) treatment was lysed in cosedimentation buffer containing 1% SDS, which yields monomeric occludin [18], and subjected to sucrose gradient centrifugation. Note that monomeric occludin and Zo-1 appear in smaller molecular weight fractions than in Figure 4D.
(TIF)

Figure S2 **Distributions of EGFP-tagged TJ proteins.** Monolayers established from stable transfectants with EGFP-Occludin (8C6, 8E9) and EGFP-claudin-1 (9B8) were stained with rhodamine-phalloidin (red) and Hoechst (blue). Images were collected as a Z-series, and then deconvoluted and overlayed to display a single composite projection. Bar: 10 mm.
(TIF)

Movie S1 **Dynamic behavior of GFP-actin in a MDCK monolayer exposed to 300 µM capsaicin.** Time-lapse series (51 frames, 15 s intervals acquired at 37°C from 16 min to 29 min after the addition of capsaicin) obtained from a stable GFP-actin transfectant using a Leica AF6000 microscope.
(MPG)
