# Supplementary figures and images for: The Reversible Increase in Tight Junction Permeability Induced by Capsaicin Is Mediated via Cofilin-Actin Cytoskeletal Dynamics and Decreased Level of Occludin
Source: PLoS One. 2013 Nov 18;8(11):e79954. doi: 10.1371/journal.pone.0079954 (PMC3832373; doi:10.1371/journal.pone.0079954)

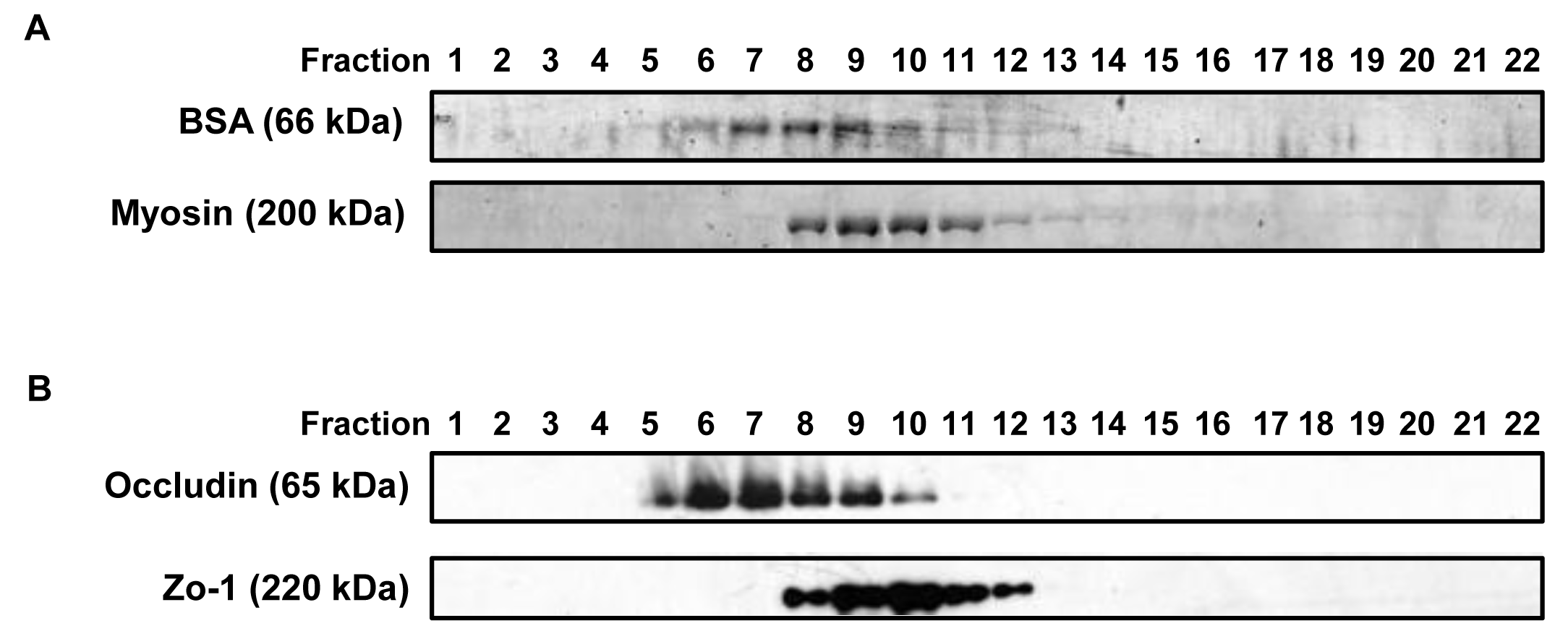

Supplement: Figure S1 — Distributions of monomeric standards and TJ proteins. (A) BSA (Sigma) and myosin (prepared from rabbit muscle) were dissolved in the cosedimentation lysis buffer and loaded on sucrose gradient for velocity gradient centrifugation. Fractions were analyzed by Coomassie Brilliant Blue staining. (B) A control monolayer without dithiobis(succinimidyl propionate) treatment was lysed in cosedimentation buffer containing 1% SDS, which yields monomeric occludin [18], and subjected to sucrose gradient centrifugation. Note that monomeric occludin and Zo-1 appear in smaller molecular weight fractions than in Figure 4D. (TIF) [file pone.0079954.s001.tif]

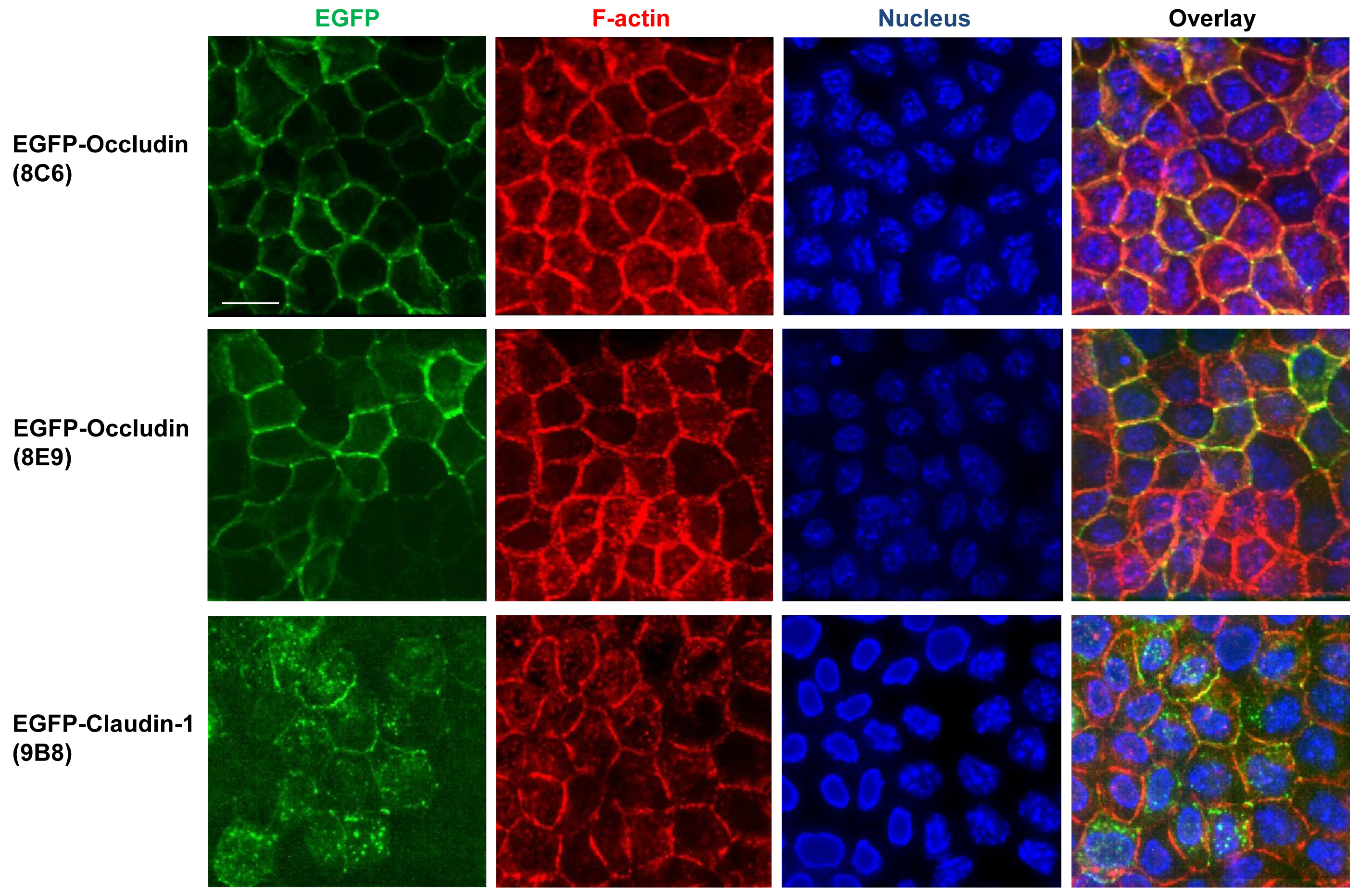

Supplement: Figure S2 — Distributions of EGFP-tagged TJ proteins. Monolayers established from stable transfectants with EGFP-Occludin (8C6, 8E9) and EGFP-claudin-1 (9B8) were stained with rhodamine-phalloidin (red) and Hoechst (blue). Images were collected as a Z-series, and then deconvoluted and overlayed to display a single composite projection. Bar: 10 mm. (TIF) [file pone.0079954.s002.tif]
